# Medical Reform

**Published:** 1877-10

**Authors:** 


### Medical Reform.

The Boston Journal opens its semi-annual mouth. It doesn't like the College association; rather scorns it, in fact. In the weary waste of ignorance which floods the medical educational world it spies but a small bit of an olive-branch Philadelphiawards. It is a pity that the Boston Journal does not mix more with its fellows. It would then gain something of a human heart, and probably take a wiser view of medical affairs. The individual reform in the schools of which it speaks, upon which only it thinks solid success can rest, is no doubt an excellent thing. Harvard is the only school, however, which as yet has succeeded with it; and that after how many years of struggle! We mistake if any thing great comes out of a movement in the University of Pennsylvania. Philadelphia is not the most favorable soil for the higher education. With all its wealth, the counter has ten times greater attraction for its young men than has the class-room. The "University," in letters at least, has not amounted to much. It has too many "college-men" in jackets. The Girard institution, too, smacks not only of yellow soap, but of infidelity. The fame of the University of Pennsylvania in medicine was gained under the old regime.

While individual reform is making its necessarily slow progress, there is a great mass of humanity which the College Association can affect speedily. Even if it do not go to the bottom of affairs, it can do much by its widespread influence. It deserves a hearty support; and people occupying the "higher planes" will make themselves a great deal more agreeable if they will accord this.—*Louisville Med. News.*

